# Low Frequency *ALK* Hotspots Mutations In Neuroblastoma Tumours Detected By Ultra-deep Sequencing: Implications For ALK Inhibitor Treatment

**DOI:** 10.1038/s41598-018-37240-z

**Published:** 2019-02-18

**Authors:** Niloufar Javanmardi, Susanne Fransson, Anna Djos, Rose-Marie Sjöberg, Staffan Nilsson, Katarina Truvé, Per Kogner, Tommy Martinsson

**Affiliations:** 1 Department of Pathology and Genetics, The Sahlgrenska Academy, University of Gothenburg, Sahlgrenska University Hospital, Gothenburg, Sweden; 2 Bioinformatics core facility, The Sahlgrenska Academy, University of Gothenburg, Gothenburg, Sweden; 3 Department of Women’s and Children’s Health, Karolinska Institutet, Stockholm, Sweden

## Abstract

The *ALK* tyrosine kinase receptor is oncogenically activated in neuroblastoma. Whereas numerous *ALK* fusion genes have been reported in different malignancies, in neuroblastoma *ALK* is mainly activated through point mutations. Three hotspot residues (F1174, F1245, and R1275) account for 85% of mutant *ALK* seen in neuroblastoma. In a cohort of 105 Swedish neuroblastoma cases of all stages, these hotspot regions were re-sequenced (>5000X). *ALK* mutations were detected in 16 of 105 patients (range of variant allele fraction: 2.7–60%). Mutations at the F1174 and F1245 hotspot were observed in eleven and three cases respectively. *ALK* mutations were also detected at the I1171 and L1240 codons in one tumor each. No mutations were detected at R1275. Sanger sequencing could confirm *ALK* status for all mutated samples with variant allele fraction above 15%. Four of the samples with subclonal *ALK* mutation fraction below this would have gone undetected relying on Sanger sequencing only. No distinct mutation spectrum in relation to neuroblastoma tumours genomic subtypes could be detected although there was a paucity of *ALK* mutations among 11q-deleted tumors. As *ALK* mutations status opens up an excellent opportunity for application of small molecule inhibitors targeting ALK, early and sensitive detection of *ALK* alterations is clinically important considering its potential role in tumour progression.

## Introduction

Neuroblastoma (NB), the most common extracranial solid cancer in childhood, displays unique heterogeneity in terms of both genomic and clinical behavior^1^, ranging from children with complete spontaneous tumour regression to children with wide spread metastatic disease resistant to treatment. Although there is treatment available for high-risk NB cases, long-term survival for this patient group is less than 50% despite aggressive treatment. The adverse outcome of high-risk NB constitutes a major clinical problem, mainly attributed to insufficient means to treat refractory or relapsed disease^2^. Consequently, one of the most important practical utilities of studying NB tumour heterogeneity lies in its implications for improving therapeutic strategy and ultimately, increased survival.

In-depth molecular characterization of cancer specimens can provide prognostic or diagnostic information and identify molecular therapeutic targets^3^. Importantly, not all mutations detected in a cancer cell are helpful for increasing knowledge regarding the mechanism of transformation. Whereas some genetic defects provide selective advantage for cancer development and/or progression, others are mere passengers without functional relevance and thus, not valuable as targetable candidates^4^. In NB, only a limited number of recurrent somatic mutations have been reported, these include mutations in *ALK* (Anaplastic Lymphoma Kinase), and a set of genes involved in chromatin-remodelling and neuritogenesis. Apart from the hotspot mutations in *ALK* most variants identified are private^5,6^. Activating germline mutations in *ALK* are the major cause of hereditary NB although somatically acquired *ALK* alterations are observed in 6–12% of sporadic cases^7–9^. Activating point mutations are mainly seen at three “hot spot” residues; F1174 (mutated to L, S, I, C or V), F1245 (mutated to L, I, V, or C), and R1275 (mutated to Q or L), all localized within the kinase domain of *ALK* and together accounting for 85% of all mutant *ALK* in NB^10^. Less frequent observed are substitutions at I1170 (to N or S) and I1171 (to N)^11^. Besides activating missense mutations, *ALK* can also be activated by amplification or rare translocation events, further supporting the necessity of this tyrosine kinase receptor for NB tumourigenesis^9,12,13^. The recent discovery of the ALK ligand, *ALKAL2* (FAM150B/AUGα),^14,15^ with a genetic locus in the genomic proximity of *ALK*, makes it tempting to speculate a role for ligand-dependent activation of the wildtype *ALK* receptor in NB pathogenesis. This makes ALK an attractive therapeutic target in NB. A number of small molecule ALK inhibitors are currently under clinical or preclinical investigation for treatment of ALK-positive malignancies. Single drug treatment with the small molecule ALK/MET inhibitor crizotinib has shown very promising results in adult non-small cell lung cancer and in large cell anaplastic lymphoma that harbour *ALK* translocations^16^. However, inhibition of *ALK* mutation in the context of the full-length receptor is complex, and crizotinib has proven to be less clinically effective in treatment of NB^10,17^. Biochemical studies of *ALK* mutations have shown that the different variants display both variable kinase activity and variable inhibitor susceptibility^10^ but this could possibly be surmountable through more high affinity inhibitors recently developed^11,18^. Second and third generation ALK specific inhibitors such as ceritinib (LDK378)^19^, brigatinib (AP26113)^20,21^, alectinib (CH-5424802)^22^, and lorlatinib (PF-06463922)^23,24^ might significantly improve NB treatment options. We recently reported on a child with underlying Fanconi anemia (FA) and ALK mutant high-risk NB responding strongly to precision therapy with the ALK TKI ceritinib^25^.

More sensitive detection of *ALK* mutations, either already present at time of diagnosis or acquired during disease progression, is of major clinical importance for NB patients as this may lead to new therapeutic possibilities^26,27^. However, only a few comprehensive studies have explored the genomic landscape of relapsing NB^28–30^. We recently showed that *ALK* alterations are enriched at relapse and that these mutations can be detected as minor subclones in the primary tumour that subsequently emerge as the major relapse clone^31^. It is highly likely that these initial subclones present at diagnosis confer a selective advantage after first line treatment with chemotherapy, ultimately leading to clonal expansion and tumour relapse. These data strongly suggest that precise molecular characterization of *ALK* mutations should be included in clinical diagnostics of NB tumours not only at diagnosis but also continuously throughout the clinical management of the patient.

To investigate this rigorously we have now undertaken a study of a series of 105 NB tumours to explore *ALK* copy number gain as well as the frequency and type of *ALK* mutations that may remain undetected using Sanger-based sequencing methodology. To do this we have employed deep parallel DNA sequencing techniques that allow detection of very low frequency of mutations and we compare this method with previously used Sanger sequencing methodology. We further intend to define the subclonal heterogeneity of the tumour and their potential role in clonal evolution and progression of NB.

## Materials and Methods

### Patient material

NB tumour samples were obtained through surgery/biopsying after informed consent from parents/guardians. The tumours were graded according the international NB staging system (INSS) and international NB risk group (INRG). All samples belong to a Swedish cohort of patients with NB tumours of all stages, Table 1. Tumours were included in the study only if containing >50% tumour cells as judged by Single Nucleotide Polymorphism (SNP) array (Affymetrix Inc., Santa Clara, CA) or through histopathological examination.

**Table 1:** Patients used in the study, ALK mutations and clinical data.

Case	ALK mut. status - Sanger	ALK mut. status - Deep seq (% of mutated allele)	Effect on ALK	Outcome	SNP array genomic profile	aad (age at diagnosis)	INRG
1	Not done	Neg		NED > 5 y	Other segm.	18	L
2	Neg	Neg		DOD	NMA	27	M
3	Neg	Neg		DOD	11q-del	76	L
4	Not done	Neg		NED > 5 y	17q-gain	14	L
5	Neg	Neg		NED > 5 y	17q-gain	102	L
6	Not done	Neg		NED > 5 y	Num only	32	L
7	Neg	Neg		NED > 5 y	Other segm.	12	L
8	Neg D1160D	Neg		NED > 5 y	Num only	21	L
9	Neg	Neg		DOD	NMA	19	M
10	Neg	Neg		DOD	Num only	48	M
11	Not done	Neg		NED > 5 y	11q-del	30	L
12	F1174L	F1174L (24,7%)	GOF	DOD	NMA	15	M
13	Neg	Neg		NED > 5 y	Num only	30	L
14	Neg	Neg		NED > 5 y	17q-gain	19	L
15	Neg	Neg		DOD	11q-del	80	L
16	Neg	Neg		DOD	NMA	18	M
17	F1174C	F1174C (19%)	GOF	NED > 5 y	Num only	25	L
18	Neg	I1171T (2,7%)	GOF	NED > 5 y	NMA	29	M
19	Neg	Neg		DOD	NMA	37	L
20	Neg	Neg		NED > 5 y	Other segm.	21	L
21	Neg	Neg		NED > 5 y	11q-del	9	L
22	Neg	Neg		DOD	11q-del	80	M
23	Neg	Neg		DOD	NMA	8	L
24	Neg	Neg		NED > 5 y	NMA	35	L
25	Neg	Neg		NED > 5 y	Num only	43	L
26	F1245I	F1245I (53%)	GOF	AWD	Other segm.	5	L
27	Neg	Neg		NED > 5 y	Num only	0	L
28	Neg	Neg		DOD	NMA	21	M
29	Neg	Neg		NED > 5 y	11q-del	74	M
30	Neg	Neg		DOD	11q-del	6	M
31	Neg	Neg		NED > 5 y	11q-del	43	M
32	Neg	Neg		DOD	17q-gain	10	L
33	Not done	Neg		NED > 5 y	Other segm.	12	L
34	Neg	Neg		DOD	11q-del	170	L
35	Neg	Neg		DOD	NMA	37	M
36	Neg	Neg		NED > 5 y	Other segm.	4	L
37	Neg	Neg		NED > 5 y	Other segm.	63	L
38	F1245C	F1245C (52,4%)	GOF	NED > 5 y	17q-gain	92	M
39	Neg	Neg		NED > 5 y	Num only	11	L
40	1st Neg/2nd F1147S	2nd F1174S (58,7) and F1174I (7,7%)	GOF & GOF	DOD	17q-gain	12	M
41	Not done	Neg		NED > 5 y	17q-gain	6	L
42	F1174I	F1174I (22%)	GOF	NED > 5 y	Other segm.	27	L
43	F1174L	F1174L (22%)	GOF	NED > 5 y	Other segm.	90	M
44	Neg	Neg		DOD	NMA	12	M
45	Not done	Neg	Amp.	DOD	NMA ( + ALK-amp)	11	M
46	Neg	Neg		DOD	Other segm.	33	M
47	Neg	F1245I (14%)	GOF	DOD	NMA	21	M
48	Neg	Neg		DOD	17q-gain	55	M
49	Not done	Neg		NED > 5 y	NMA	15	M
50	1st Neg /2nd F1174L	F1174L (25,9%)	GOF	DOD	NMA	29	M
51	Not done	Neg		NED > 5 y	11q-del	33	M
52	Neg	Neg		DOD	11q-del	32	M
53	F1174I, const.	F1174I (61,2%)	GOF	DOD	Other segm.	1	M
54	Neg	Neg		DOD	Num only	0	MS
55	Neg	Neg		DOD	11q-del	21	M
56	Neg	Neg		DOD	NMA	14	M
57	Neg	Neg	Amp.	DOD	NMA ( + ALK-amp)	31	M
58	Not done	Neg		NED > 5 y	11q-del	10	L
59	Not done	Neg		NED > 5 y	17q gain	98	L
60	Neg	Neg	Amp.	DOD	NMA ( + ALK-amp)	25	M
61	F1174L	F1174L (18%)	GOF	DOD	NMA	41	L
62	Neg	Neg		NED > 5 y	11q-del	92	M
63	Not done	Neg		NED < 5 y	Other segm.	6	L
64	Neg	Neg		NED < 5 y	Other segm.	5	L
65	Not done	Neg		NED < 5 y	NMA	28	M
66	Not done	Neg		NED < 5 y	Other segm.	32	L
67	Not done	Neg		NED < 5 y	Num only	2	L
68	Not done	Neg		NED < 5 y	17q gain	37	L
69	Not done	Neg		DOD	NMA	39	M
70	Neg	Neg		DOD	NMA + 11q-del	28	M
71	Not done	Neg		NED < 5 y	NMA	18	L
72	Neg	Neg		NED < 5 y	11q-del	18	L
73	Not done	Neg		NED < 5 y	11q-del	26	L
74	Not done	Neg		NED < 5 y	Other segm.	11	M
75	Neg	Neg		NED < 5 y	11q-del	70	L
76	Not done	Neg		NED < 5 y	Num only	1	L
77	Not done	Neg		NED < 5 y	Num only	18	L
78	Neg	Neg		NED < 5 y	Other segm.	0	L
79	Neg	Neg		DOD	NMA + 11q-del	30	M
80	Neg	Neg		NED < 5 y	Other segm.	37	L
81	Neg	Neg		NED < 5 y	NMA	13	L
82	Neg	Neg		NED < 5 y	NMA	10	L
83	Neg	Neg		NED < 5 y	11q-del	18	L
84	Neg	F1174L (15,1%)	GOF	NED < 5 y	NMA	23	M
85	Neg	Neg		NED < 5 y	Num only	6	L
86	Neg	Neg		NED < 5 y	11q-del	158	M
87	Neg	Neg		NED < 5 y	Other segm.	12	L
88	Neg	Neg		NED < 5 y	NMA	33	M
89	Neg	Neg	Amp.	NED < 5 y	NMA ( + ALK-amp)	39	M
90	Neg	Neg		NED < 5 y	Num only	2	L
91	Neg	Neg		DOD	NMA + 11q-del	11	M
92	Neg	F1174L (14%)	GOF	NED < 5 y	17q gain	37	L
93	Neg	Neg		NED < 5 y	11q-del	124	L
94	Neg	Neg		NED < 5 y	11q-del	51	L
95	Neg	Neg		NED < 5 y	11q-del	13	L
96	Neg	Neg		NED < 5 y	11q-del	29	L
97	F1174L	F1174L (22,1%)	GOF	NED < 5 y	NUM only	8	L
98	Neg	Neg		NED < 5 y	11q-del	50	M
99	L1240V	L1240V (57,4%)	GOF	DOD	17q gain + chrom.tr	44	M
100	Not done	Neg		NED < 5 y	Other segm.	2	L
101	Not done	Neg		NED < 5 y	NMA	27	L
102	Not done	Neg		NED < 5 y	17q-gain	66	M
103	Not done	Neg		NED < 5 y	Num only	8	L
104	Not done	Neg		NED < 5 y	NMA	15	L
105	Not done	Neg		AWD	NMA	37	M

*MYCN* status and tumour copy number aberrations has been determined earlier, using SNP arrays and/or FISH^32^. Clinical data and genomic profiles of included patients are summarized in Table 1. Patients were treated according to relevant treatment protocols. Ethics approval of treatment protocols was obtained according to national guidelines and the study was authorized by the local ethical committees (Karolinska Institutet and Karolinska University Hospital). The methods were carried out in accordance with the relevant guidelines and regulations.

Following DNA extraction using standard procedures, mutations of the receptor tyrosine kinase (RTK) domain of the *ALK* gene were analyzed by two different deep parallel sequencing methods (i) the HaloPlex™ target enrichment system and (ii) an amplicon-based assay for sequencing of three hotspot regions in *ALK* (exon 23, 24 and 25) using Illumina MiSeq sequencing platform (Illumina, San Diego, CA). In order to ensure the potential of our method in detection and discrimination between *ALK* three hotspot residues F1174, F1245 and R1275, three serial dilutions (undiluted, 1:10, and 1:40) from each of the three hotspot amplicons (*ALK* exon 23, 24, 25) were generated using 20 ng DNA of three patients each carrying the relevant mutation and dilute it with 20 ng of unmatched germline DNA. Subsequently, eight germline genomic DNAs from healthy donors and three tumours with known *ALK* mutation served as controls to quantify background abundances.

### HaloPlex™ target enrichment system

HaloPlex (Agilent Technologies, Santa Clara, CA) is a custom in-solution capture hybridization method that was used for deep sequencing of *ALK* gene. Custom probe design for the *ALK* region of interest (exon 21–25) was created with SureDesign (Agilent). In brief, 225 ng of genomic DNA from each sample were fragmented using eight seperate restriction enzyme digestion reactions and denatured prior hybridization to the HaloPlex probe library. The HaloPlex Target Enrichment System Protocol (Version D.5) was followed without modification to perform the library preparation and target capture using the HaloPlex Exome capture baits. We assessed library quality using Agilent’s 2200 TapeStation system. Enriched and indexed library were prepared for 150-bp paired-end sequencing (2 × 150 bp) on an Illumina MiSeq sequencing system (Illumina, San Diego, CA).

### Amplicon-based assay for targeted sequencing of relevant *ALK* exonic regions

For sequencing library construction, 12 ng of genomic DNA were used to amplify the *ALK* regions of interest containing hotspots F1174 (exon 23), F1245 (exon 24), and R1275 (exon 25). During the PCR, using KAPA HiFi HotStart DNA Polymerase (Kapa Biosystems, Wilmington, MA), the region-specific primers including Illumina sequencing adapters overhang were used. After amplicon purification with Ampure magnetic beads (Beckman Coulter Inc., Brea, CA), dual indices are attached using Nextera XT Index Kit (Illumina). Following another clean-up and quality control, the final libraries were pooled equimolarly, denatured prior pair-end sequencing (2 × 150 bp) using MiSeq v.3 reagent kit (Illumina). The number of libraries sequenced per flow cell was adjusted to achieve a depth of coverage of at least 5000x for the amplicons in each sample.

### Detection of variants

**Figure 1:**
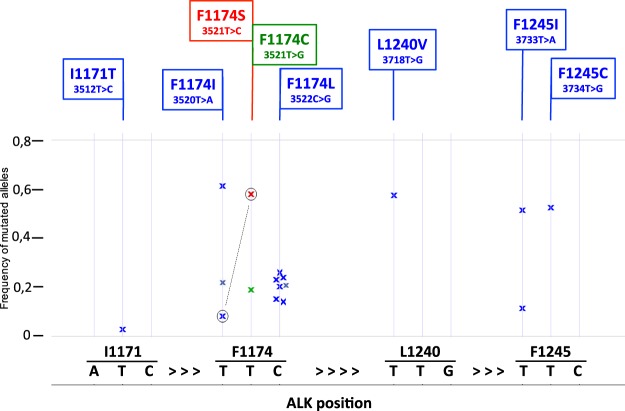
*ALK* mutations in neuroblastoma patients. Frequency distribution of mutated *ALK* allele at the *ALK* F1245, L1240, F1174 and T1171 hotspots detected in 16 samples. The x-axis represents the genomic coordinates of the targeted region, the y-axis represents the percentage of high-quality reads supporting the mutated base. The base corresponding to the reference genome sequence is reported below the graph, for the forward strand. The mutated base as well as the amino acid change from the reference is indicated above the graph. The circeled-x represent the same tumuor that showed two different mutations in *ALK*.

Using MultiQC^33^, quality of reads was assessed through Fastqc showing that majority of reads never dropped below a phred score of 30 at any position (Supplementary Figure 1). Demultiplexing of pooled libraries was performed using the unique indexes introduced during sample preparation. All reads were quality trimmed and adopters were cut with Cutadapt^34^. Paired reads (2 × 150 bp) were mapped against Human genome build hg19 (Human Genome Browser, http://genome.ucsc.edu/; hg19) with BWA-MEM default settings^35^. We did variant calling with the tool UnifiedGenotyper with the option to EMIT_ALL_CONFIDENT_SITES offered in the Genome Analysis Toolkit (GATK)^36^. This option was used to be able to predict low frequency alleles, since it gives the allele count for each allele at each position (Supplementary Table 1). We focused on hg19 coordinates exon 21 chr2:29445383-29445473, exon 22 chr2:29445210-29445274, exon 23 chr2:29443572-29443701, exon 24 chr2:29436850-29436947, and exon 25 chr2:29432652-29432744 (Human Genome Browser; hg19), to analyze mutation status of *ALK* tyrosine kinase domain. The most frequently observed sporadic *ALK* substitutions at amino acids positions F1174 (exon 23), F1245 (exon 24) and R1275 (exon 25) were studied in detail (Fig. 1).

### Data analysis

**Figure 2:**
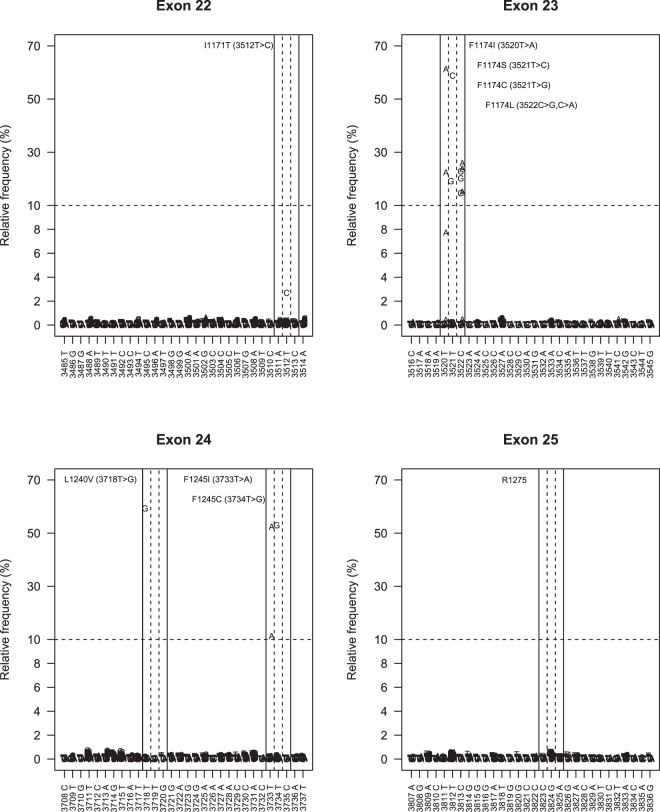
Analysis of detection level and background noise. Considerations concerning the problem of detection of low frequency alleles. Frequency distribution of mutated *ALK* allele at the *ALK* exon 21 to 25 detected in 16 samples. The *x*-axis represents the coding sequence (CDS) position of the targeted region (the base corresponding to the reference genome sequence is reported below the graph, for the reverse strand), the *y*-axis represents the fraction of high quality reads supporting each base. Sequencing analysis reveals the presence of the *ALK* mutation with a statistically significant difference from the background variability. The lowest level of mutation detected were in exon 22 for case #18, where the level of mutated alleles were 2,7%. As seen in this figure this low frequency differs distinctly from the background level.

In order to evaluate the level of signal vs. noise in the mutation detection system, mutations were determined by visually inspecting nucleotide frequencies at relevant hotspots (Fig. 2). Nucleotide frequencies larger than five standard deviations from background noise were classified as mutations.

The datasets analysed during the current study are available from the corresponding author on request.

## Results

**Figure 3:**
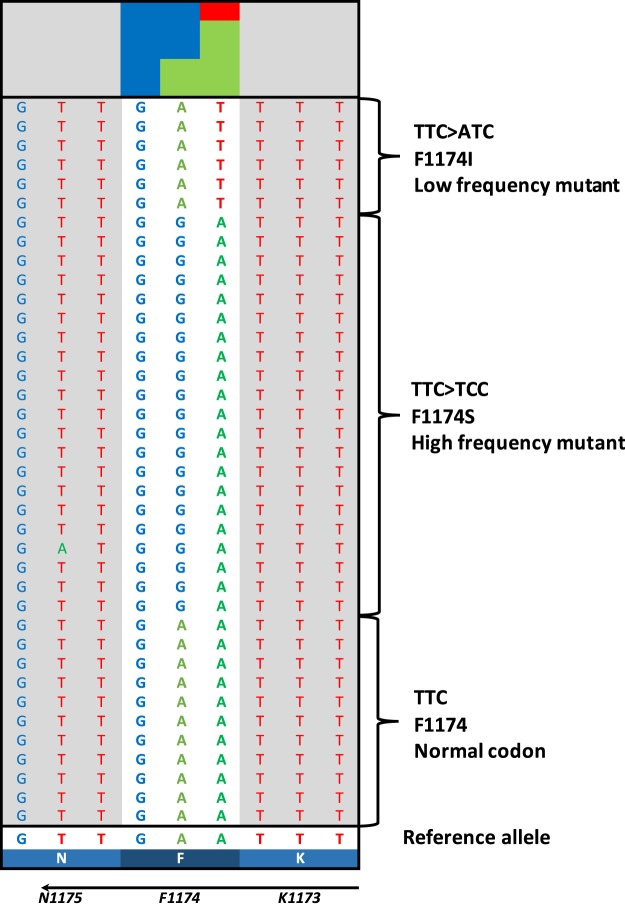
NB case with two mutations. Aligned sequencing reads visualized in the Integrative Genomics Viewer DNA reference standards with *ALK* wildtype and two positive A > G (F1174S, MAF 58%) and A > T (F1174I, MAF 8%) variant calls at cis and trans alleles observed in one NB patient. MAF, mutant allele frequency.

In this study we performed ultra-deep sequencing in order to determine the frequency of *ALK* hotspot mutations that might be missed with conventional Sanger sequencing due to different degrees of sensitivity. This was done through using two different sequencing strategies, either the hybridization-based HaloPlex target enrichment method or amplicon-sequencing, in a series of totally 105 NB samples. Total aligned reads across overlapping amplicons translated into a minimum coverage depth per position of 5000x (range 5000–65000x). The percentages of bases at coordinates that correspond to positions of *ALK* hotspots mutations were studied in detail. The background variation frequency at the relevant coordinates was determined in the controls and compared to cases followed by analysis of the mutational status of *ALK* exon 21–25 in the hybridization-based assay and exon 23–25 in the amplicon-based assay. *ALK* mutations with a variant allele frequency less than 20% were detected in six tumours while ten other cases showed *ALK* mutations with higher variant allele frequency (between 20–60%). At residue I1171 in the *ALK* locus (chr2:29445213), an *ALK* variant was detected in one case with the frequency of mutated allele as low as 2.7%. This was the lowest variant frequency level detected by deep-targeted sequencing in our cohort. At the F1174 hotspot (chr2:29443695-29443697), alterations were observed in eleven cases: seven cases harbored a mutation leading to the amino acid change F1174L, two cases with F1174I, one each of the F1174C, and F1174S substitutions, with the mutated allele fractions ranging from 14% to 60%. Interestingly the patient harbouring F1174S mutation with 58% frequency also exhibited a subclone with an F1174I mutation detected at 8% frequency of the mutated allele (Fig. 3). At the F1245 hotspot (chr2:29436858-29436860), alterations were detected in 3 tumours: two samples showed a F1245I mutation while the third case carried the F1245C mutation, with frequencies of 14%, 51% and 52%, respectively. At the L1240 locus (chr2:29436875) a L1240V substitution were detected in one patient with a mutated allele fraction at 57%. In total, sixteen out of the 105 tumour samples (15.2%) were *ALK* mutant positive (Fig. 1, Table 1). We have not observed any mutations at hotspot mutation site R1275. No additional variants outside previously reported mutational sites were observed in the targeted regions in our cohort.

**Figure 4:**
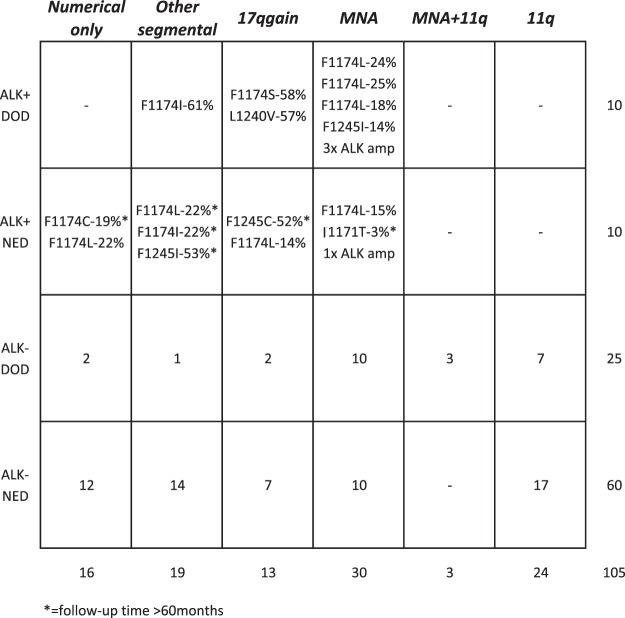
The NB tumours presenting mutations sorted by genome profile and outcome. Note that most *ALK* mutations are present in the *MYCN* amplified cases while no 11q cases showed *ALK* mutations in this set. Also the four cases with *ALK* amplification are included.

**Figure 5:**
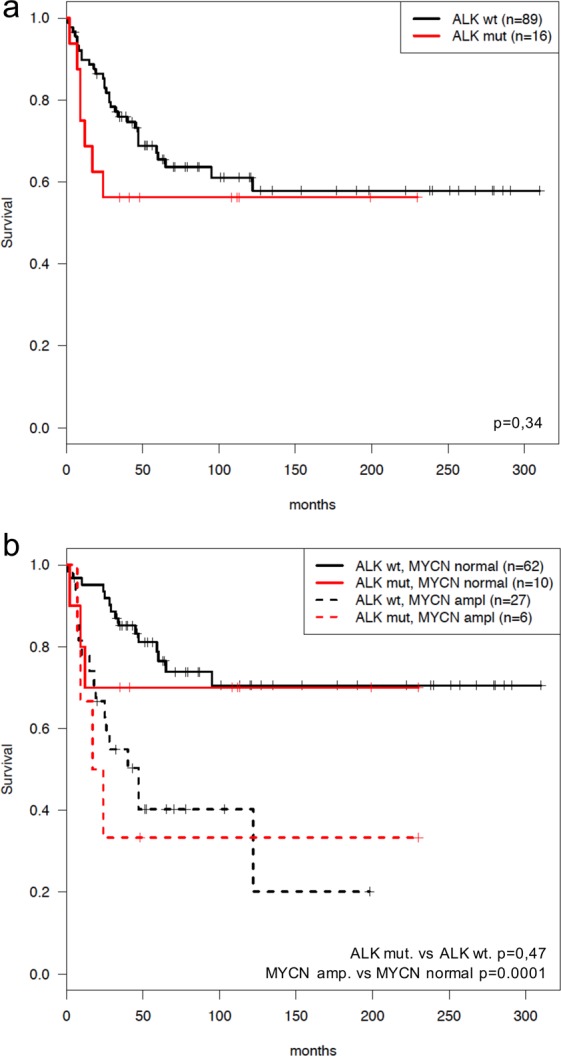
Kaplan-Meier survival comparisons of cases with *ALK* mutation vs non-*ALK* mutation cases. Kaplan-Meier (overall survival) analysis according to the presence or absence of somatic *ALK* aberrations in 105 neuroblastoma tumours (**a**) *ALK* mutated versus non-*ALK* mutated cases (**b**) OS according to *MYCN* and *ALK* status (*MYCN* amplification vs. no amplification; *ALK* alteration versus no alteration). *ALK* mutational status does not add prognostic information to the independent clinical parameter *MYCN* status. Pos, positive for *ALK* alteration (*ALK* mutations and/or amplification). Wt, wildtype. OS, overall survival.

*ALK* mutations were observed in tumors from all clinical stages. No distinct preference of F1174 or F1245 mutations in relation to different genomic subtypes (numerical only, other segmental, 17q-gained or MYCN-amplified) could be identified (Fig. 4). However, no *ALK* mutations were detected in any of the tumours, in this set, carrying segmental 11q-deletion while in patients without 11q deletion, 20% presented *ALK* point mutations (n = 27; P value 0.01; Fig. 4). No statistical difference in overall survival was observed when comparing NB patients whose tumor harbour an *ALK* mutation with cases without an *ALK* mutation. The comparison of survival of patients with *ALK* wild-type or *ALK* mutation, with or without *MYCN* amplification showed a poorer survival in patients whose tumours harbour *MYCN* amplification, with or without *ALK* mutation (Fig. 5). The presence of the F1174 *ALK* mutation was not found to have an influence on survival when comparing patients with *ALK* F1174 mutated tumours versus all other patients (data not shown).

In this study, tumour samples with a mix of up to 50% normal cells were included which may underestimate the mutation frequencies of heterogeneous tumour samples. Thus it would be expected that subclonal mutation events would represent a greater fraction of the tumour cell population in the actual tumour mass as compared to analyzed DNA fragments. In this regard, our detection of ten *ALK* variants with mutated allele fraction above 20% might be interpreted as clonal, whereas the six *ALK* mutations observed with a fraction below 20% might be interpreted as subclonal events that might be unrecognized through Sanger sequencing. All *ALK* mutated samples were also tested by Sanger sequencing which confirmed all *ALK* mutations occurring with a mutated allele fraction above 15% leaving four samples with a mutated allele fraction below 15% undetected by Sanger sequencing in our hands.

In addition, four of the 105 tumours included also had *ALK* gene amplification detected from previously performed SNP microarrays. All four cases also had co-amplification of *MYCN* and no *ALK* mutation was detected in any of these samples. Focal low copy number gain for *ALK* was also detected in one *MYCN*-amplified patient who also had a chromothripsis pattern for chromosome 2 (case #57). Partial chromosome 2p gain that includes the *ALK* locus was observed in 26 patients while gain of whole chromosome 2 was seen in 11 cases. Interestingly, all patients (n = 4**)** with partial 2p gain that also carry an *ALK* mutation are dead of disease (Table 1). In the tumours with partial 2p gain, the extra chromosomal segments varied in size from 25 to 130 Mb and included both *ALK* and *MYCN* except in 6 patients (data available on request). In these six cases, the *MYCN* amplification segment was present with a breakpoint proximal to the start genomic position of gained region. The partial 2p gains encompassing the *ALK* locus were present in only 4 of 16 mutated tumours indicating that 2p gain is not a common mechanism for increasing mutated *ALK* copy number.

## Discussion

With the emergence of targeted therapeutic strategies, full characterization of genetic alterations becomes crucial to improve treatment and patient outcome. Intratumour genetic heterogeneity has been reported in number of human malignancies and provides insights into clonal evolution and pathogenesis^37,38^. This type of heterogeneity may thereby hinder therapeutic strategies that depend on results from single tumour-biopsy samples and provides a risk to render clinically important variants undetected if using less sensitive detection methods.

In order to determine the frequency of *ALK* mutations in exon 23, 24 and 25 including those also present at subclonal level, 105 diagnostic NB samples were analyzed through ultra-deep sequencing that provides increased sensitivity to that of conventional Sanger sequencing (Table 1). Ultra-deep sequencing (>5000x) enables discovery of rare variants present at frequencies as low as 1%. In this study, we employed two different strategies, the HaloPlex enrichment kit (Agilent) and an amplicon PCR-based approach, both of which were sequenced on a MiSeq Illumina platform. Coverage, uniformity and variant calling ability did not differ between the methods that were evaluated in parallel on a subset of tumours in our cohort. The results are excellent with low overall background noise in both techniques although the HaloPlex method is somewhat more costly and slightly more labor intensive. The amplicon approach requires less DNA as compared to HaloPlex (12 ng vs. 225 ng) and also easier processing when analyzing few samples per run that might be more common in a clinical setting. Other approaches can be applied; Positive mutant–specific amplification can be done by Droplet Digital PCR (ddPCR) which is able to detect a mutant allele frequency of less than 1%.

*ALK* point mutations were detected in 16 samples while gene amplification was identified in an additional four samples, giving a total of *ALK* mutational events in 20 of 105 NB samples (19%). Variant allele fraction of observed *ALK* point mutations in this study ranged from 2.7% to 60% of all NB cases, with a subclonal (mutated allele fraction <20%) observed in 6 of 16 *ALK*-mutated samples. This is in concordance with the work of Bellini *et al*. who performed deep sequence of exons 23 and 25 of *ALK* in a series of 276 NB samples and showed that more than half of the identified point mutations were subclonal events that might have gone undetected by Sanger sequencing. This highlights the importance of deep sequencing techniques for the identification of mutations in NB, especially when targeted therapy is a clinical consideration^31,39^. All of the NB -associated *ALK* mutations, observed in our dataset are known to confer strong gain of function effect to ALK *in vivo* assays, and are therefore known as drivers in the progression of NB tumours^10,11,40^. The potent ligand-independent F1174L mutation was present in 7 cases of which 4 had MYCN-amplification whereas the other three displayed other genomic profiles. Three of four patients with *MYCN* amplification and *ALK* F1174L co-occurrence showed adverse outcome (Table 1, Fig. 4) suggesting a cooperative effect between the two aberrations. It has previously been suggested that the F1174L mutation might contribute to a particular growth advantage for *MYCN*-amplified NBs^41,42^. We observed that the majority of *MYCN*-amplified tumours (4 of 6) contained subclonal (<20% variant allele fraction) *ALK* aberrations while in *MYCN*-nonamplified tumours the majority of *ALK* mutations (8 of 10) were clonal. This implies that *MYCN*-nonamplified tumours are dependent on the presence of this constitutive active kinase in a higher percentage of the cell population. In addition to mutations, ALK activation can also result from high-level gene amplification. In keeping with previous studies, amplification of *ALK* almost exclusively occurs in *MYCN*-amplified tumours^43^. Interestingly, we do not detect any mutations among the 11q-deleted genomic subgroup of NB tumours (Fig. 4) in this set of tumours. Finally, we show that chromosome 2p is not frequently gained in tumours with *ALK* mutations, indicating that mutated *ALK* alleles are not selected for high expression by copy number gain. However, 2p gains are present in as much as 19.3% of all NBs with segmental imbalances^44^. This raises the possibility that NB tumours with 2p gain may instead benefit from extra copies of *MYCN* and *ALK* wildtype receptor in collaboration with its recently described ligand, *ALKAL2* located at 2p25.

This study of 105 NBs shows that *ALK* mutations can be observed in tumours of various clinical stages and of various genomic profiles. We have previously shown through ultra-deep sequencing that subclonal *ALK* mutations can be detected in NB tumours at diagnosis and that they are enriched at relapse, suggesting that *ALK* alterations may contribute to progression and a more aggressive disease^31^. This is further supported by another study showing that NB patients with tumours that harbor *ALK* alterations have a decreased 5-year overall survival as compared to those with wildtype *ALK*^39^. We fail to show any statistically significant *ALK*-dependent difference in overall survival in our material (Fig. 5) but this could be due to smaller sample size in our study. Interestingly, we observed a case containing at the same time both F1174S and F1174I mutations with 58% and 8% allele frequencies respectively. Our group has previously performed Sanger sequence analysis for this patient revealing a homozygous missense mutation for F1174S^45^. At this time, the deep sequencing revealed a heterozygous F1174S mutation cooperating with a secondary subclone harbouring another amino acid substitution, I instead of S, for the same locus (Fig. 3). This case provides evidence that extra information can be achieved with NGS methods that are impossible to gain by Sanger approach alone. In the near future, additional information emerging from these deep sequencing studies will be useful for guiding treatment decisions.

Treating NB is still a therapeutic challenge despite recent advances in pediatric oncology. However, due to the recognition of oncogenic forms of ALK as driver in NB malignancy, ALK have emerged as a tractable therapeutic target. Small molecule ALK inhibitors may become the gold standard therapy in NB treatment, making diagnostic high sensitive detection of *ALK* mutations a necessary step in identifying optimal treatment modalities. We recently reported a NB patient with novel *ALK*-I1171T variant who revealed complete clinical remission upon treatment with ceritinib^25^. However, a large-scale analysis of the spectrum of *ALK* mutations, their clinical significance, and their biochemical properties in NB is essential to direct preclinical and clinical studies of ALK inhibitors and to identify patients likely to benefit from ALK inhibition in NB. In our previous study^31^ we showed that some NB tumours have subclones harbouring *ALK* mutations at diagnosis that may contribute to tumour evolution and to relapse. Thus, deep-targeted sequencing would enable us to monitor changes in clonal and subclonal composition of cancer cells during disease progression to orient treatment decisions and to benefit of the patient.

Despite the encouraging development of next generation ALK inhibitors, both intrinsic and acquired resistance may occur when using single drug treatment for receptor tyrosine kinase inhibitors. One mechanism of resistance is acquirement of secondary genetic changes that reduce the accessibility of the hydrophobic inhibitor-binding pocket for small molecule inhibitor by increasing the affinity for ATP instead. A number of these resistant promoting gatekeeper mutations have been demonstrated in non-small cell lung cancer patients treated with crizotinib or alectinib^46,47^. The recent discovery of the ALK ligand, *ALKAL2* (FAM150B/AUGα)^14,15^ with a genetic locus in the genomic proximity of *ALK* and *MYCN*, may aid in further development of therapeutic tools for intervention in NB development in the near future.

## Conclusion

*ALK* is the most frequently mutated gene in NB and has provided new hope for NB treatment using ALK-specific inhibitors. As *ALK* mutations may contribute to disease progression and relapse, early detection and eradication of these subclones is of uttermost importance as they may improve patient prognosis. We show here that through ultra-deep sequencing, *ALK* point mutations were detected in 16 samples whereof 6 were subclonal that may remain undetected through conventional sequencing methods. This proves that ultra-deep sequencing could become crucial in clinical analysis as it allows sensitive detection of activating *ALK* mutations as well as of resistance promoting mutations present at subclonal level.

## Supplementary information

Supplementary dataset
