# Supplementary material for: Low Frequency ALK Hotspots Mutations In Neuroblastoma Tumours Detected By Ultra-deep Sequencing: Implications For ALK Inhibitor Treatment
Source: Sci Rep. 2019 Feb 18;9:2199. doi: 10.1038/s41598-018-37240-z (PMC6379392; doi:10.1038/s41598-018-37240-z)
Supplement: Supplementary file 1 — Supplementary dataset [file 41598_2018_37240_MOESM1_ESM.pdf]

Low Frequency *ALK* Hotspots Mutations In Neuroblastoma Tumours Detected By Ultra-deep Sequencing: Implications For ALK Inhibitor Treatment

Niloufar Javanmardi, Susanne Fransson, Anna Djos, Rose-Marie Sjöberg, Staffan Nilsson, Katarina Truvé, Per Kogner and Tommy Martinsson

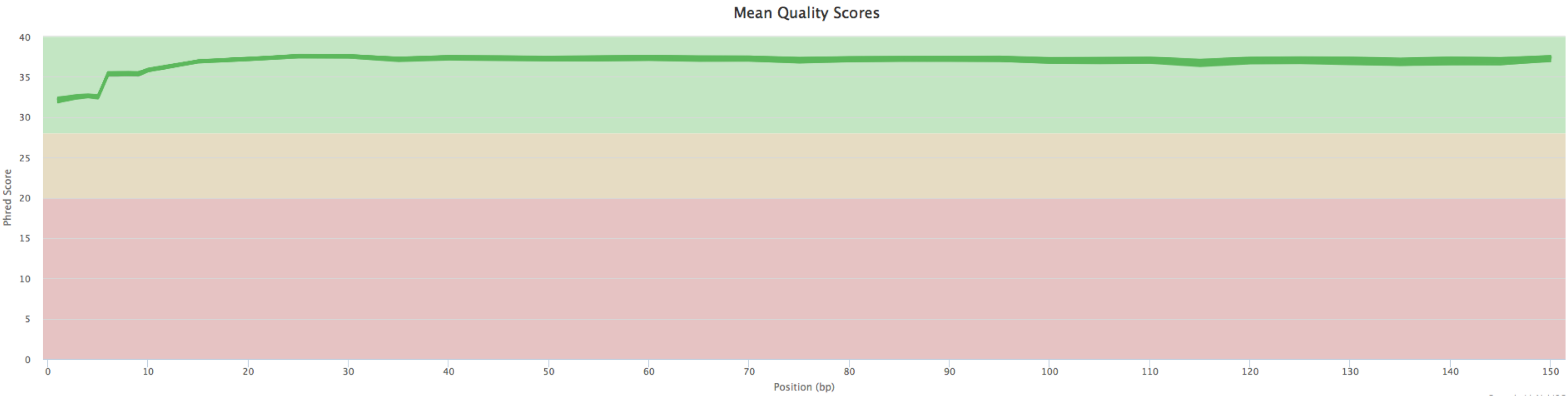

Supplementary figure 1. Quality assessment for DNA- sequencing reads. Median of Phred quality score distribution over all reads across all samples in each base (i.e., sequencing cycle). There is one graph per each patient based on the relating Fastqc file. X-axis shows reads position. Y-axis shows mean quality score. A quality score of 10, 20, or 30 means that the probability that the base is called wrong is 10%, 1%, or 0.1%, respectively.

Low Frequency *ALK* Hotspots Mutations In Neuroblastoma Tumours Detected By Ultra-deep Sequencing: Implications For ALK Inhibitor Treatment

Niloufar Javanmardi, Susanne Fransson, Anna Djos, Rose-Marie Sjöberg, Staffan Nilsson, Katarina Truvé, Per Kogner and Tommy Martinsson

Position	Ref	Case #12					Case #17					Case #18					Case #38				
		A	C	G	T	Depth	A	C	G	T	Depth	A	C	G	T	Depth	A	C	G	T	Depth
29432663	T	14	29	22	65313	65378	0	2	8	8811	8821	1	2	3	5400	5406	3	15	18	52617	52653
29432664	C	48	65261	16	52	65377	14	8794	4	9	8821	2	5393	3	6	5404	29	52555	15	53	52652
29432665	G	25	8	65281	57	65371	3	0	8818	1	8822	0	1	5399	4	5404	43	10	52534	60	52647
29436858	G	3	4	26290	12	26309	3	2	9988	5	9998	0	0	9217	2	9219	32	7	42700	29	42768
29436859	A	26247	46	6	8	26307	9989	2	4	2	9997	9213	6	1	0	9220	20262	22464	9	33	42768
29436860	A	26275	10	13	8	26306	9986	2	5	3	9996	9214	2	2	1	9219	42696	36	25	7	42764
29436873	C	18	26269	2	6	26295	2	8791	0	3	8796	2	8104	2	0	8108	29	42712	5	9	42755
29436874	A	26928	9	10	4	26951	7958	1	1	0	7960	6900	2	0	0	6902	43123	32	6	2	43163
29436875	A	26920	11	5	7	26943	6247	0	1	0	6248	5409	1	0	1	5411	43088	24	4	7	43123
29443695	G	22	8	32801	10808	43639	1	0	9029	3	9033	0	0	7029	0	7029	10	6	39941	65	40022
29443696	A	42828	108	31	49	43016	6906	1634	2	3	8545	6659	0	3	5	6667	39571	95	14	32	39712
29443697	A	42821	153	9	27	43010	8525	4	11	3	8543	6659	3	2	3	6667	39592	83	10	24	39709
29445212	G	3	2	5141	2	5148	11	2	8464	12	8489	4	0	5492	5	5501	3	0	5364	2	5369
29445213	A	5121	9	10	7	5147	8470	4	8	7	8489	5369	5	121	5	5500	5358	1	6	5	5370
29445214	T	2	2	10	5133	5147	4	1	8	8476	8489	0	1	5	5495	5501	2	0	3	5365	5370

Supplemental table 1. Patients used in the study, ALK mutations and clinical data

The mean depth of coverage achieved by MiSeq Illumina deep-sequencing of *ALK* targeted regions. Original variant calls are shown for 4 mutated cases.
